# Bimetallic Bismuth‐Based Nanoparticles From Pseudo‐Tetrahedral Zintl Anions

**DOI:** 10.1002/smll.73221

**Published:** 2026-03-25

**Authors:** Megan A. Parker, Dirk Hauschild, Ravi Priya, Constantin Wansorra, Ralph Steininger, Benjamin Peerless, Lothar Weinhardt, Clemens Heske, Stefanie Dehnen

**Affiliations:** ^1^ Institute of Nanotechnology (INT) and Karlsruhe Nano Micro Facility (KNMF) Karlsruhe Institute of Technology (KIT) Karlsruhe Germany; ^2^ Institute For Photon Science and Synchrotron Radiation (IPS) Karlsruhe Institute of Technology (KIT) Karlsruhe Germany; ^3^ Institute For Chemical Technology and Polymer Chemistry (ITCP) Karlsruhe Institute of Technology (KIT) Karlsruhe Germany; ^4^ Department of Chemistry and Biochemistry University of Nevada Las Vegas (UNLV) Las Vegas Nevada USA

**Keywords:** bimetallics, bismuth, nanoparticles, precursors, soft and hard X‐ray photoelectron spectroscopy, Zintl salts

## Abstract

While Zintl compounds comprising Bi‐based anions of p‐block elements have been used as starting materials for larger bismuth‐based clusters, their potential to form nanoparticles has not yet been explored. Here, bimetallic nanoparticles are synthesized from the oxidation of bismuth‐based pseudo‐tetrahedral Zintl anions (InBi_3_)^2−^, (Sn_2_Bi_2_)^2−^, (TlBi_3_)^2−^, and (Pb_2_Bi_2_)^2−^. The anions rapidly oxidize at ambient conditions to form metallic seeds, with the mild oxidizing agent PVP (polyvinylpyrrolidone), which also serves as a stabilizing agent for nanoparticle growth. Each elemental combination behaves uniquely, resulting in bimetallic nanoparticles of varying forms (i.e., alloyed, core‐shell, and *Janus*‐type). The resulting nanoparticles show a relatively narrow size distribution with median diameters of ∼20–25 nm and exhibit ultraviolet (UV) absorption, with spectral features tunable by composition. The morphology and composition were analyzed by scanning transmission electron microscopy (STEM), high‐resolution transmission electron microscopy (HRTEM), micro‐X‐ray fluorescence spectroscopy (µ‐XFS), powder X‐ray diffraction (PXRD), synchrotron‐based hard and soft X‐ray photoelectron spectroscopy (HAXPES and PES), and attenuated total reflectance—Fourier transform infrared spectroscopy (ATR‐FTIR). This approach demonstrates that binary Zintl anions can serve as versatile molecular precursors for designing heterometallic nanoparticles with controlled composition, morphology, and optical properties.

## Graphical abstract

Bismuth‐based Zintl anions are explored as precursors for synthesizing bimetallic nanoparticles with tunable compositions and morphologies, including alloyed, core–shell, and *Janus* types. The resulting nanoparticles display composition‐dependent UV absorption. This study highlights Zintl anions as versatile molecular building blocks for controlled fabrication of heterometallic nanoparticles with customizable optical and structural properties.

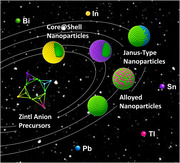

## 1 Introduction

Plasmonic nanoparticles manipulate light at the nanoscale, enabling applications in photocatalysis, sensing, photothermal therapy, and optoelectronic devices [1, 2]. Noble metal nanoparticles, such as gold and silver nanoparticles, have long dominated plasmonic research owing to their well‐defined and tunable localized surface plasmon resonances (LSPRs) in the visible and near‐infrared regions. Compositional engineering through alloying or doping has further proven to be a powerful strategy for tuning the optical properties of these materials [3, 4, 5]. Extending precise spectral control into the ultraviolet (UV) region, however, requires the development of alternative plasmonic materials. Among promising candidates, post‐transition metals (Al, Ga, In, Sn, Tl, Pb, and Bi) exhibit LSPR features in the UV range that can be readily tuned by varying the nanoparticle composition, size, and shape [6]. In particular, bismuth (Bi) nanoparticles, thanks to their interband plasmonic behavior in the UV region, offer considerable potential for distinctive optical properties in this region [7].

Conventional synthetic routes producing metallic nanoparticles—such as solvothermal methods, laser ablation, or other high‐energy approaches—have enabled the production of homometallic Bi nanoparticles with controllable size and morphology [8, 9]. However, reports on Bi‐based heterometallic nanoparticles, or, more broadly, mixed‐metal pnictogen‐based nanoparticles, remain scarce [10, 11, 12, 13, 14]. This gap underscores the need for alternative synthetic routes that offer further structural and compositional control, enabling precise tuning of the optical behavior.

Bi‐based Zintl anions as molecular precursors represent a largely unexplored platform for the bottom‐up design of new heterometallic nanomaterials. Several examples of nanomaterials produced from Zintl anions derived from lighter main group elements (Sn, Te, Ge, and Si) exist. For example, tetrahedral Si_4_^4−^ or Ge_4_^4−^ clusters or larger nine‐atom cages have been used as reactive precursors for nanoparticles with controllable size [15, 16, 17, 18, 19, 20, 21]. Surfactant‐driven self‐organization of Ge_9_^4−^ clusters produced a novel hexagonal nanoporous Ge phase [22]. Moreover, solution‐phase self‐assembly of Sn_4_^4−^ clusters resulted in inorganic/organic composite nanomaterials, while co‐assembly of Sn_9_^4−^ and Ge_9_^4−^ clusters enabled the fabrication of mixed‐metal Sn/Ge thin films [23, 24, 25]. Despite these advances, only a single example involving the synthesis of homometallic Bi nanoparticles via the extraction of Bi anions from a Na–Bi alloy has been reported [26], highlighting an untapped opportunity to use these clusters as molecular building blocks for heterometallic nanoparticles.

Here, we demonstrate that Bi‐based bimetallic nanoparticles can be produced from the oxidation and self‐assembly of (InBi_3_)^2−^, (TlBi_3_)^2−^, (Sn_2_Bi_2_)^2−^, and (Pb_2_Bi_2_)^2−^ Zintl anions. Unlike conventional nanoparticle syntheses, which typically rely on separate redox agents and stabilizers at elevated temperatures, these highly reactive anions undergo spontaneous oxidation and nucleation upon dropwise addition of a stabilizer under ambient conditions. The resulting particles self‐assemble into ∼20–25 nm bimetallic nanostructures during this one‐pot, room‐temperature process. To our knowledge, this represents the first example of multimetallic nanoparticles produced directly from mixed‐metal Zintl anion precursors. Four distinct bimetallic architectures are obtained, varying from alloyed to phase‐segregated morphologies. The crystallinity, phases, and elemental compositions of the nanoparticles were examined from the core to the shell via scanning transmission electron microscopy (STEM), high‐resolution transmission electron microscopy (HRTEM), micro‐X‐ray fluorescence spectroscopy (µ‐XFS), powder X‐ray diffraction (PXRD), attenuated total reflectance‐Fourier transform infrared spectroscopy (ATR‐FTIR), and synchrotron‐based hard (HAXPES) and soft (PES) X‐ray photoelectron spectroscopy (together abbreviated as ‘(HAX)PES’), while UV‐visible spectroscopy reveals composition‐dependent LSPRs in the UV region. This synthetic route from binary Zintl anion precursors at room temperature provides access to a variety of Bi‐based nanostructures, with morphology and composition dictated by precursor stoichiometry and the intrinsic tendency of Bi to intermix with different constituent elements.

## 2 Results and Discussion

### 2.1 Overview of the Synthesis of Bi‐Based Bimetallic Nanoparticles

**FIGURE 1:**
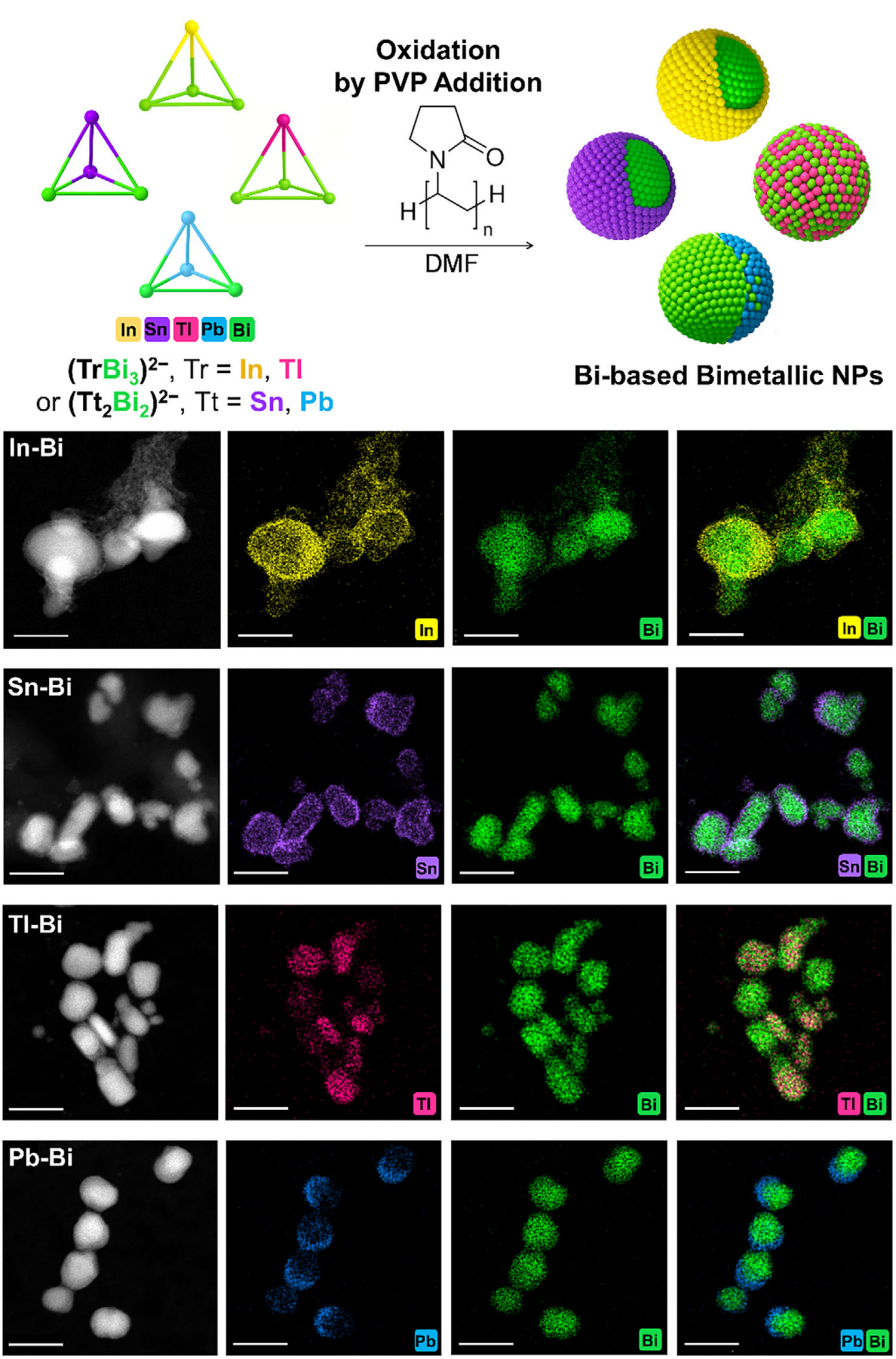
(Top) Synthesis scheme of Bi‐based bimetallic particles from the oxidation of pseudo‐tetrahedral Zintl anions. (Bottom) High‐angle annular dark‐field STEM images and elemental mapping of the In–Bi, Sn–Bi, Tl–Bi, and Pb–Bi nanoparticles. All scale bars represent 50 nm.

Binary Bi‐Zintl anions oxidize readily under mild conditions, forming larger Zintl anions, metallic powders, and, as demonstrated here for the first time, bimetallic nanoparticles [27, 28, 29]. In this study, we find that the addition of a high molecular‐weight polymer to solutions of binary Zintl anions is oxidizing enough to form zero‐valent metallic clusters. When dissolved in DMF, the (InBi_3_)^2−^ and (TlBi_3_)^2−^ salts form green solutions, while the (Sn_2_Bi_2_)^2−^ and (Pb_2_Bi_2_)^2–^ salts form red‐brown solutions. Initially, PVP (polyvinylpyrrolidone, M_n_ ≈ 40,000 g/mol) was added alongside a mild oxidizing agent, benzophenone. However, the addition of benzophenone led to rapid oxidation, producing metallic powder that immediately precipitated from solution. In contrast, when PVP was used alone, a significant fraction of the precursor remained in solution, which was the method of choice thereupon. Within minutes of dropwise PVP addition, the solutions darken, indicating the formation of metallic seeds. Thus, PVP *simultaneously* serves as a stabilizer and as a mild enough oxidant, enabling a facile, one‐pot synthesis of bimetallic nanoparticles at room temperature. We attribute this oxidation to residual impurities or trace amounts of water that are difficult to fully remove, even after overnight drying of the polymer under vacuum, with the measured water content of the PVP solution being <10 ppm. The different elemental combinations of the explored precursors form core–shell (for In‐Bi and Sn‐Bi), alloyed (for Tl–Bi), or *Janus*‐type (for Pb–Bi) nanoparticles (Figure 1). In each case, the nanoparticles are 20–25 nm in diameter (as determined by measuring 300 particles in TEM images, Figure S1).

### 2.2 Nanoparticle Characterization

#### 2.2.1 In–Bi Nanoparticles

To synthesize the In‐Bi nanoparticle precursor, the (InBi_3_)^2−^ anion is extracted from the ternary Zintl phase K_5_In_2_Bi_4_ with ethane‐1,2‐diamine (*en*) in the presence of crypt‐222, in which co‐crystallization of larger heterometallic species (In_4_Bi_5_)^3−^ as its [K(crypt‐222)]^+^ salt has also been reported as a minor product [30]. Here, we explored the oxidation of (InBi_3_)^2−^ with PVP all the way to the nanoscale and find that both In and Bi are retained in individual nanoparticles, but within each particle elemental diffusion leads to the formation of Bi‐rich cores, whereas In diffuses out toward the outermost 2–3 nm of the nanoparticles, as determined by TEM (Figure 1). This formation of core–shell structures from a single anionic precursor differs from previous reports of Bi‐based core–shell nanoparticles synthesized via polyol routes, where Bi^3^^+^ ions are first reduced to form elemental bismuth cores, followed by reduction of co‐precursor ions at the particle surface to generate shells [11].

**FIGURE 2:**
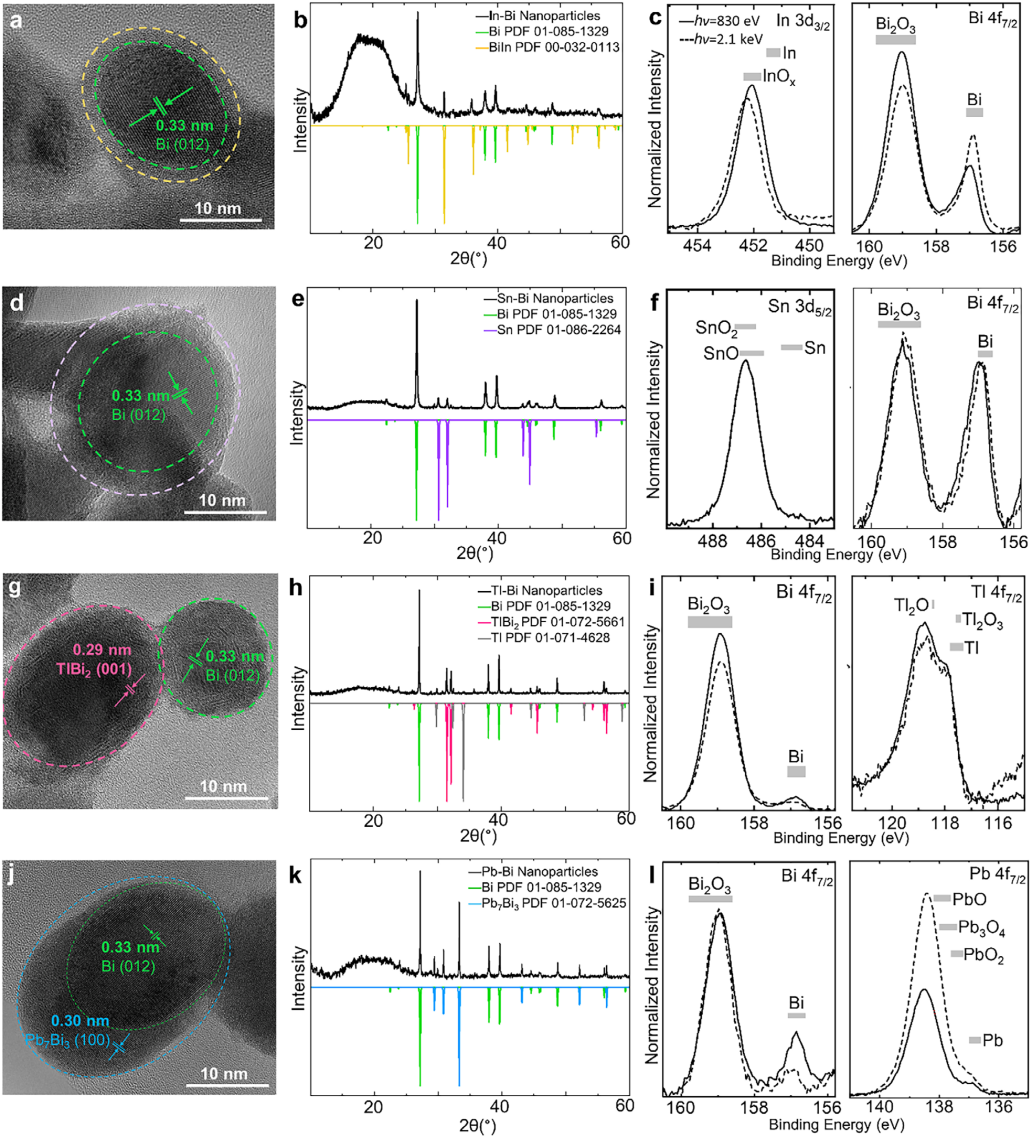
HRTEM images, PXRD patterns [in comparison with the powder diffraction files (PDF) from the Inorganic Crystal Structure Database (ICSD)] [31], and high resolution (HAX)PES spectra of the In–Bi (a–c), Sn–Bi (d–f), Tl–Bi (g–i), and Pb–Bi (j–l) nanoparticles. PES spectra (*hν* = 830 eV) are shown with solid lines and HAXPES spectra (*hν* = 2.1 keV) are shown with dashed lines. Literature binding energy ranges for different bulk compounds are given as gray bars above each spectrum [32].

Overall, the nanoparticle composition measured by µ‐XFS showed In at a slightly higher ratio than the parent Zintl anion, with an In:Bi ratio of 1:2.2 (±0.5) (Figure S2). It is hypothesized that Bi metal precipitates rapidly during nanoparticle formation, as the In : Bi ratio in the discarded pellet was enriched in Bi, with a measured ratio of 1:4.3 (±0.5). Lattice fringes corresponding to metallic Bi are observed in the cores of the nanoparticles via HRTEM imaging, while the outer shells of the particles are amorphous (Figure 2a). From PXRD patterns (Figure 2b), metallic Bi reflections, minor peaks corresponding to BiIn alloy, as well as a broad peak centered around 2θ of ∼20° were observed. Coherent domain lengths for the metallic Bi phase were calculated from the PXRD patterns and found to be ∼0.7 nm (±0.1), much smaller than the overall particle size (∼20 nm) (Table S1). The coherent domain lengths suggest that the Bi cores are polycrystalline; however, strain, amorphous contributions, and/or polymer background (Figure S3) give rise to uncertainty in this conclusion. The broad peak corresponds to an amorphous phase, which could be a result of amorphous Bi or In, metal‐oxides, or organic residues at the nanoparticle surface.

To derive the chemical environment of In and Bi at the surface of the particles, the In 3d_3/2_ and Bi 4f_7/2_ (HAX)PES detail spectra (Figure 2c) and In MNN X‐ray excited Auger electron spectroscopy (XAES) measurements (Figure S6) are analyzed. In 3d_3/2_ (instead of In 3d_5/2_) was chosen to minimize the energetic overlap with the Bi 4d_5/2_ core level. We find the In 3d_3/2_ peaks at ∼452.2 and 452.1 eV for photon energies of 2.1 keV and 830 eV, respectively. The In MNN is very weak, likely due to In being covered by adsorbed remainders of the solvents used during synthesis. The M_4_N_45_N_45_ Auger transition appears at ∼406.5 eV. Taken together, the In 3d_3/2_ core level and Auger spectra indicate an oxide‐like chemical environment [31], confirming that In is fully oxidized. The Bi 4f_7/2_ core level spectra show that Bi is also present at the (sub)surface in two main chemical environments, with peaks centered at ∼157.0 and 159.0 eV that can be assigned to metallic Bi and Bi oxide, respectively. The metallic Bi component increases with respect to the Bi oxide component for the measurement with higher photon excitation energy (2.1 keV), that is, with more bulk‐sensitive probing. The peak‐area ratios “Bi‐oxide”/(“Bi‐oxide”+“Bi‐metal”) [Bi‐O/(Bi‐O+Bi‐M)] were calculated and found to decrease from 0.82 to 0.72 (±0.02) when going to higher photon energies (Figure S7), indicating more metallic Bi in the nanoparticle's interior (as expected from the elemental maps in Figure 1). Together with the TEM and PXRD data, these results suggest that the In‐Bi nanostructures possess a few nanometers thick shell primarily composed of mixed In‐ and Bi‐oxides.

#### 2.2.2 Sn–Bi Nanoparticles

Core‐shell nanostructures similar to the In–Bi nanoparticles were observed when the (Sn_2_Bi_2_)^2−^ anion was oxidized with PVP. Previous studies have shown that the mild oxidation of (Sn_2_Bi_2_)^2−^ can yield larger binary Zintl anions, such as (Sn_7_Bi_2_)^2−^ or (Sn_4_Bi_4_)^2−^ [29], suggesting that intermediate Sn‐Bi assemblies may form during the initial stages of oxidation. Here, as oxidation proceeds and nanoparticles form, it is suspected that the lighter Sn atoms diffuse outward, forming a distinctive shell (∼2–3 nm in thickness, as determined by TEM) surrounding crystalline Bi cores (Figure 1). The nanoparticle composition determined by µ‐XFS was approximately equivalent to the Zintl anion, with a 1:1.1 (±0.5) Sn: Bi ratio (Figure S2). HRTEM images revealed only lattice fringes with d‐spacings corresponding to metallic Bi in the cores (Figure 2d), whereas PXRD patterns also indicated the presence of a minor β‐Sn crystalline phase in the bulk sample (Figure 2e). Coherent domain lengths for the metallic Bi phase within the Sn‐Bi nanoparticles were calculated from the PXRD patterns and found to be ∼0.8 nm (±0.1), evidencing the likely polycrystallinity within the larger, ∼20 nm, Bi cores (Table S1). A broad, less intense peak centered at 2θ of ∼20° also shows the presence of an amorphous phase.

To investigate the surface of the particles, the Sn 3d_5/2_ and Bi 4f_7/2_ core‐level regions were analyzed using (HAX)PES. The binding energy of the Sn 3d_5/2_ signal measured at 830 eV photon energy (486.6 eV) indicates that Sn is oxidized at the surface of the nanoparticle (Figure 2f) [28]. The corresponding spectral region at 2.1 keV photon energy was not analyzed due to an energetic overlap with the Si KLL Auger feature. The Bi 4f_7/2_ spectra measured at both photon energies show Bi in two distinct Bi environments, with binding energies of 157.0 and 159.1 eV, which we again assign to metallic Bi and Bi oxide, respectively (see corresponding gray boxes for literature values in Figure 2f). In this case, the Bi‐O/(Bi–O + Bi–M) ratio remains nearly constant (0.59 ± 0.02) for the two excitation energies (Figure S7). This might indicate a thicker Bi oxide shell in comparison to the In‐Bi nanoparticles, consistent with a higher Sn:Bi precursor ratio in comparison to the In:Bi precursor ratio.

#### 2.2.3 Tl–Bi Nanoparticles

In comparison to In, several more examples of Tl‐ and Bi‐containing Zintl anions have been reported (as [K(crypt‐222)]^+^ salts), obtained upon treatment of (TlBi_3_)^2−^ with d‐ or f‐block metal compounds—in some cases as byproducts of Zintl clusters including the d‐ or f‐block metal atoms [33, 34, 35]. The tendency to include Tl in larger anionic clusters has been found to be more successful due to the preference of Bi atoms in clusters to bond to heavier elements with similar relative charges and size. The oxidation of (TlBi_3_)^2−^ with PVP resulted in some particles comprised of only Bi, and other particles made up of both Tl and Bi. HRTEM and PXRD identified the Tl‐Bi alloy as the intermetallic phase TlBi_2_ (Figure 2g,h). The mismatch in Tl:Bi ratio between the stable alloy (1:2) and the precursor (1:3) results in an excess of Bi during the oxidation of the Zintl anions and the formation of two separate phases. The near‐instantaneous formation of the TlBi_2_ intermetallic alloy at room temperature in this system, likely due to the similar chemistry of the constituent elements, contrasts with previous reports of Bi‐based intermetallic nanoparticles (with Rh, Ir, and Pt) that typically require elevated temperatures and stepwise reduction to first form intermediates [12, 13, 14].

The elemental composition measured by µ‐XFS yielded a Tl:Bi ratio of 1:2.5 (±0.5) in the bulk nanoparticle sample (Figure S2). The low interfacial wettability between these phases leads to the formation of separate single‐phase nanoparticles [36]. Coherent domain lengths were calculated and averaged for both the Bi and TlBi_2_ phases and found to be 2.0 and 1.4 nm (±0.1 nm), respectively, appearing to indicate polycrystallinity in the much larger, ∼25 nm nanoparticles in both types of nanoparticles (Table S1).

The Bi and Tl 4f_7/2_ (HAX)PES spectra each show two distinct chemical environments (Figure 2i), with peaks at ∼118.0 and 118.8 eV for Tl and ∼156.9 and 158.9 eV for Bi, and a Tl:Bi ratio of 1:3.3 (±0.5). The ratio of metals at the surface closely matches that of the metals within the precursor used. Bulk reference values for metallic and oxide environments for both elements are plotted above the respective peaks; however, no PES literature data are available for the TlBi_2_ intermetallic phase that we observe in the PXRD patterns. Having a mixture of alloyed and Bi‐only nanoparticles makes it difficult to draw detailed conclusions, but we do observe a decrease of the Bi–O/(Bi–O + Bi–M) ratio from 0.97 to 0.92 (±0.02) when going to higher photon energies, showing a higher fraction of metallic Bi in the nanoparticle interior (Figure S7).

#### 2.2.4 Pb–Bi Nanoparticles

Oxidation of the (Pb_2_Bi_2_)^2−^ anion also leads to metal‐alloying and phase separation in the nanoparticles, giving rise to two distinct phases: metallic Bi and a Pb–Bi alloy. In this case, the two phases partially wet each other so that the Pb–Bi alloy forms a crescent‐shaped cap around the metallic bismuth cores. HRTEM images and PXRD indexing identify the intermetallic phase as Pb_7_Bi_3_ (Figure 2j,k). Both phases are observed in the PXRD pattern, confirming uniformity in the bulk sample. The Pb:Bi ratio of the nanoparticle sample, determined by µ‐XFS, matches that of the Zintl anion precursor at 1:1.1 (±0.5) (Figure S2). Coherent domain lengths were calculated and averaged over both the Bi and Pb_7_Bi_3_ phases and found to be 1.6 and 1.9 nm (±0.1 nm), respectively, appearing to indicate polycrystallinity of both phases in the different regions (Table S1). The contrasting behavior between the Bi/TlBi_2_ and Bi/Pb_7_Bi_3_ systems—where the former exhibits complete phase separation into distinct nanoparticles and the latter shows partial wetting within individual particles—can be attributed to differences in the interfacial energies between the coexisting phases and their interactions with the surrounding medium. The degree of phase wetting that leads to *Janus*‐type nanoparticle formation is well‐documented in metal–polymer *Janus* nanoparticles [37, 38].

The Bi and Pb 4f_7/2_ (HAX)PES detail spectra each show at least two distinct chemical environments (with peaks at ∼136.9 and 138.5 eV for Pb and peaks at ∼156.9 and 159.0 eV for Bi); literature values for bulk metal and various metal‐oxides are shown above the spectra (Figure 4e) [32]. Higher quantities of Pb were detected compared to Bi (Pb:Bi = 1:0.3 (±0.1)), consistent with the Pb‐rich alloy crescent caps on individual nanoparticles observed in the TEM images. Again, to our knowledge no PES literature data are available for the Pb_7_Bi_3_ intermetallic phases observed in our PXRD, so we cannot unambiguously conclude whether the intermetallic environment is present at the nanoparticle surface. We do again observe a decrease in the Bi‐O/(Bi–O + Bi–M) ratio from 0.96 to 0.87 (±0.02) when going to higher photon energies (Figure S7), showing more metallic Bi in the nanoparticle interior.

#### 2.2.5 Organic Species Adsorption

**FIGURE 3:**
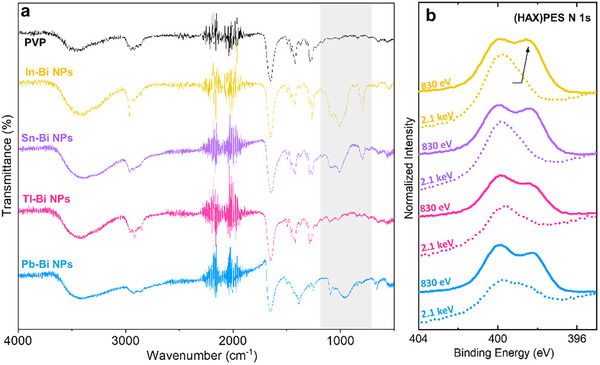
(a) FTIR‐ATR spectra: PVP in black, In–Bi in yellow, Sn–Bi in purple, Tl‐Bi in magenta, and Pb‐Bi in blue. (b) N 1s photoemission spectra for the four nanoparticle samples following the same color scheme. Solid lines represent PES spectra excited at 830 eV, and dashed lines represent HAXPES spectra excited at 2.1 keV.

The presence of C, N, and O in photoelectron spectroscopy spectra (Figure S4), coupled with FTIR‐ATR spectra, indicates some surface adsorption of the solvents and/or PVP used during synthesis. Comparison of the FTIR‐ATR spectra of the nanoparticles with that of pure PVP confirms surface coating by PVP, with the same signature peaks observed at ∼1285, 1420, and 1650, 2920, and 3460 cm^−1^ in all nanoparticle samples (Figure 3a). However, additional peaks are observed in the highlighted region, around ∼1000 and 1100 cm^−^^1^, evidencing additional C─C and C─O vibrational bands. We suspect that adsorption of organic solvents (DMF, isopropanol, toluene) used during synthesis and/or washing give rise to these vibrational modes.

The N 1s (HAX)PES spectra validate the suspected organic species adsorption. Changes in the N 1s PES spectra for different photon energies were observed, indicating differences in N adsorption at the surface and into the bulk of the nanoparticles. While the more bulk‐sensitive 2.1 keV measurements indicate a dominating nitrogen species at ∼399.9 eV, an additional nitrogen species at ∼398.3 eV is detected for the more surface sensitive PES measurement (Figure 3b). The peak present for both PES and HAXPES at ∼399.9 eV can be attributed to PVP, based on previous literature reports describing the stabilization of metal seeds by PVP during nanoparticle syntheses [39, 40]. Since the reaction medium (DMF) also contains nitrogen, DMF likely represents the secondary source of nitrogen adsorption during and/or after nanoparticle formation, present at the outermost surface of the samples.

### 2.3 Optical Properties

**FIGURE 4:**
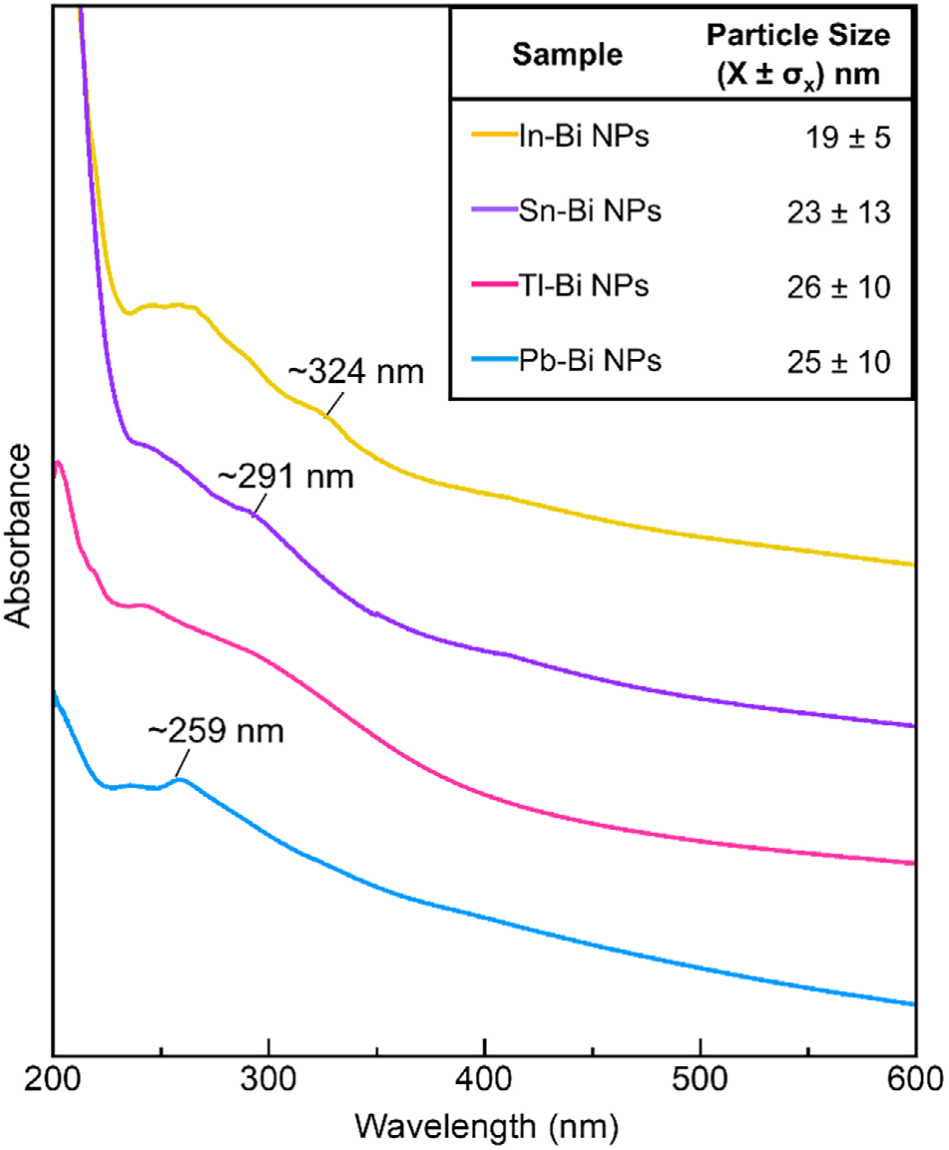
Absorption spectra of our four Bi‐based nanoparticle samples dispersed in isopropanol. The inset table summarizes the particle size distribution for the four samples.

In the UV‐vis absorption spectra, we find features in the UV region of all samples. The most distinct features appear between ∼245–260 nm, and secondary features are observed at longer wavelengths (Figure 4). Theoretical studies have shown that the dipolar LSPR mode in bismuth particles can be tuned from the near‐IR for Bi particles several hundreds of nanometers in diameter, to the UV for Bi particles of 20 nm in diameter [6, 7, 41]. The primary absorption peaks in the UV‐region observed in our four nanoparticle samples reside at similar wavelengths, but are dampened in comparison to the ones predicted by Mie theory for pure 20 nm Bi nanoparticles (Figure S8) [6, 7]. In this size regime, scattering is negligible, allowing a direct comparison of the experimental absorption spectra to theoretical spectra for homometallic 20 nm Bi nanoparticles. The observed peak dampening could be a result of several factors, such as the presence of the native oxide layer, which extends a few nanometers beneath the nanoparticle surfaces (see discussion above), variations in the dielectric environment, introduced by the polymer coating surrounding the nanoparticles, or size polydispersity, which is outlined in the inset table [42, 43]. The secondary feature to the onset absorption varies in position for the different samples. For the Pb–Bi sample, a distinct peak around 259 nm is observed, while for the Tl–Bi, a secondary shoulder blends into the onset of the absorption peak. For the Sn–Bi sample, a secondary shoulder around 291 nm is observed, which is red‐shifted for the In‐Bi sample to ∼324 nm. The secondary shoulders arising for the In–Bi and Sn‐Bi systems suggest that oxide shells have greater effects on absorption spectra in comparison to a polymer coating (which exists in the case of all four nanoparticle samples). All nanoparticle samples remain stable for several weeks, with no visible changes in their absorption spectra over time. The LSPR sensitivity to composition, surface chemistry, and nanostructure highlights the potential of these systems as versatile platforms for designing UV‐responsive plasmonic materials.

## 3 Conclusion

For the first time, it is demonstrated how heterometallic Zintl clusters can be used as precursors to form multimetallic nanoparticles, and how different elemental combinations of similarly structured Zintl anions result in varied nanostructures. The four pseudo‐tetrahedral Zintl anions based on In, Sn, Tl, and Pb—combined with Bi—were oxidized to form similarly sized, but compositionally different bimetallic nanoparticles. The different precursor stoichiometries between the Group 13/14 elements and Bi were shown to affect the final nanoparticles formed. The elemental pairs with the largest atomic mass differences (In–Bi and Sn–Bi) led to diffusion of the lighter elements from the nanoparticle core to the shell, resulting in core—shell structures with Bi predominantly concentrated in the core, and a shell composed of mixed‐metal oxides. The pairs with smaller atomic mass differences, that is, Tl and Pb, resulted in a higher degree of elemental mixing within the nanoparticles, leading to intermetallic phases and alloyed nanoparticles. Hard and soft X‐ray photoelectron spectroscopy of all sample surfaces corroborated the structural information by giving insights into the native oxide layer at the (sub)surfaces of nanoparticles. Furthermore, adsorbed polymeric species and other organic synthesis residues could be detected.

We demonstrate how a simple, one‐pot strategy with a commercial and inexpensive polymer can be used to oxidize heterometallic Zintl anions to form distinctly new and unique nanoparticles, in which the chemical composition of the particles is defined by the precursor composition. Using this method, four unique nanoarchitectures could be synthesized. The nanoparticles show LSPRs in the UV, with secondary spectral features that shift depending on chemical makeup. This highlights the use of Zintl anions as nanoparticle precursors in producing new materials with structural and compositional control that would otherwise be unattainable with traditional nanochemistry routes.

## 4 Experimental Methods

### 4.1 Materials

Precursor syntheses were performed under exclusion of air and moisture using standard Schlenk techniques and/or in Ar‐filled gloveboxes. Ethane‐1,2‐diamine (en, Aldrich, 99.8%) was distilled from CaH_2_ and stored over 4 Å molecular sieves. Crypt‐222 (Kryptofix 222, Aldrich) was dried in vacuo (∼10^−3 ^mbar) for 12 h. Ternary solids K_5_Tr_2_Bi_4_ (Tr = In, Tl) or KTtBi (Tt = Sn, Pb) were synthesized and extracted in en as described previously [30, 43]. Polyvinylpyrrolidone (PVP, MW ∼40 000, Aldrich) was dried in vacuo (∼10^−3 ^mbar) for 4 h. Dimethylformamide (DMF, 99.8%, Aldrich) was dried by three freeze‐pump‐thaw cycles and stored over 4 Å molecular sieves. Isopropanol (99%, Aldrich) and toluene (99%, Aldrich) were used as received.

### 4.2 Nanoparticle Syntheses

In an argon‐filled glovebox, ca. 60 mg of [K(crypt‐222)]_2_(TrBi_3_) or [K(crypt‐222)]_2_(Tt_2_Bi_2_) (0.040 mmol, 1.0 eq.) were weighed and added to a round‐bottom Schlenk flask equipped with a magnetic stir bar. DMF (30 mL) was added, followed by the dropwise addition of 1 g of PVP dissolved in 10 mL DMF (0.025 mmol, 0.6 eq.). The reagents were stirred with magnetic agitation at room temperature for 16 h under Argon and a brown solution was observed with metallic powder precipitate. After reaction completion, the solid solution was exposed to air, and subsequent washing steps were conducted in air. To separate the precipitate from the nanoparticles, 30 mL toluene was added to the crude product and the contents were centrifuged at 3000 g for 10 min. The nanoparticle‐containing supernatant solution was retained and the formed pellet was discarded. Afterward, the nanoparticle‐containing supernatant solution was then pelleted by centrifugation at 10000 RCF for 10 min. The nanoparticles were resuspended in a 50:50 mixture of isopropanol and toluene and centrifuged once again at 10000 RCF for 10 min. The synthesis affords the nanoparticles in approximately 50–67% yield (∼30‐40 mg) from 60 mg of precursor.

### 4.3 TEM Characterization

Particles dispersed in isopropanol were sonicated for 3 min. One drop was cast onto a gold grid covered with a carbon film for analysis by TEM (transmission electron microscopy). Bright field HRTEM images were acquired using a probe‐corrected Thermo Fischer Themis 300 electron microscope, which was operated at 300 keV. STEM images were obtained using a high‐angle annular dark‐field (HAADF) detector. Energy‐dispersive X‐ray (EDX) spectroscopy elemental maps were obtained using a Super‐X detector in STEM mode. HAADF and EDX spectral imaging were acquired and processed using Velox software.

### 4.4 Micro‐X‐Ray Fluorescence Spectroscopy (µ‐XFS)

The analysis was carried out using a HORIBA XGT X‐ray analytical microscope on dried powder samples. Measurements were conducted under vacuum conditions (1 mbar) utilizing a Rh X‐ray source operated at 50 kV and 632 µA. Fluorescence emission was recorded over a measurement time of 180 s. Quantitative analysis of In, Sn, Tl, Pb, and Bi was based on their characteristic Lα_1_ X‐ray emission lines.

### 4.5 Powder X‐Ray Diffraction

Measurements were performed on dried powder samples on a Stoe Stadi MP. A Debye‐Scherrer construction was used and the samples were measured with Cu K_α_ radiation in a 2θ‐angle from 10° to 70°. For the interpretation of the obtained diffractograms and removal of the background, the program WinXPow Version 3.5.0.2 was used.

### 4.6 Infrared Spectroscopy

Measurements were recorded on dried powder samples at ambient conditions with a Thermo Scientific NICOLET iS50 FTIR instrument, equipped with a diamond ATR single crystal.

### 4.7 Soft and Hard X‐Ray Photoelectron Spectroscopy

Particles dispersed in isopropanol were spin‐coated onto a Au‐coated Si wafer (Platypus Tech.) via 3 × 20 µL at 40 000 RPM in the Ar‐filled glovebox of the Materials for Energy (MFE) laboratory at the KIT Light Source [44]. The samples were then transferred without air exposure to the X‐SPEC beamline [45] for PES and HAXPES measurements, using the focusing variable‐line‐space plane grating monochromator (FVLS‐PGM) and the Si(111) double crystal monochromator (DCM), respectively. Spectra were collected with a SPECS Phoibos 225 electron analyzer. The binding energy was calibrated by a reference measurement of the Au 4f_7/2_ peak at a binding energy of 83.96 eV [46] and checked between measurements of different samples. Depth‐dependent information within the first few nanometers was obtained by alternating the excitation photon energy between 2.1 keV and 830 eV, thereby increasing the characteristic 1/e attenuation length *λ* for the photoelectrons from the Bi 4f state from ∼4 to ∼1–2 nm [47]. Surveys showed the following elements at both excitation energies: In, Sn, Tl, Pb, and Bi from the respective samples; Au and Si from the Au‐coated Si substrates; C, O, and N from organic solvents and reagents used during synthesis; and S, which likely originates from other chemicals commonly used in the glove box or from substrate impurities (Figure S4) [48]. Element‐specific regions were recorded and analyzed in detail, including In 3d_3/2_, In MNN, and Sn 3d_5/2_ for the respective In‐ and Sn‐containing samples. For Bi, Pb, and Tl, the 4f_7/2_ peaks were analyzed to avoid overlap of the Bi 4f_5/2_ signal with the Bi 5s and S 2p peaks. The enlarged spectral region showing the 4f peaks of Bi, Pb, and Tl (Figure S5) highlights Bi in all four samples and confirms the presence of Tl and Pb (only in their respective samples).

### 4.8 UV‐Visible Absorption Spectroscopy

Absorption spectra were recorded of dilute suspensions of nanoparticles in isopropanol (∼1 mg/1 mL) in quartz cuvettes, using the transmission mode of a Varian Cary 5000 UV‐vis‐NIR spectrometer from Agilent. The optical path length for the measurement was set to 1 cm. The spectral data were collected at a scan rate of 300 nm/min with a 1 nm data interval, using the Cary WinUV Scan Application software (version 6.2.0.1588). Prior to each measurement, a background spectrum of a blank quartz cuvette filled with isopropanol was recorded to account for solvent absorption.

### 4.9 Mie Theory Calculations

The absorption, scattering, and extinction cross‐sections of spherical Bi nanoparticles, 20 nm in diameter, were calculated using Mie theory, using freely available codes [49]. The refractive index of Bi is taken from Werner et al. [50]. The embedding medium refraction index for isopropanol used is 1.377.

## Funding

Deutsche Forschungsgemeinschaft (DFG, German Research Foundation), Collaborative Research Centre “Tracking the Active Site in Heterogeneous Catalysis for Emission Control” (CRC 1441, Project‐ID 426888090), project A07N, Collaborative Research Center “4f for Future” (CRC 1573, project number 471424360), project A4, as well as projects GZ:INST 121384/64‐1 FUGG, GZ:INST 121384/65‐1 FUGG, and GZ:INST121384/66‐1 FUGG. European Union (ERC, BiCMat, 101054577).

## Conflicts of Interest

The authors declare no conflicts of interest.

## Supporting information




**Supporting File**: smll73221‐sup‐0001‐SuppMat.pdf.

## Data Availability

The data that support the findings of this study are available from the corresponding author upon reasonable request.
