# Supplementary material for: Bimetallic Bismuth‐Based Nanoparticles From Pseudo‐Tetrahedral Zintl Anions
Source: Small. 2026 Mar 25;22(28):e73221. doi: 10.1002/smll.73221 (PMC13181514; doi:10.1002/smll.73221)
Supplement: Supplementary file 1 — Supporting File: smll73221‐sup‐0001‐SuppMat.pdf. [file SMLL-22-e73221-s001.pdf]

Bimetallic Bismuth-based Nanoparticles from Pseudo-Tetrahedral Zintl Anions

Megan A. Parker[1], *Dirk Hauschild*[2,3,4], *R. Priya*[2], *Constantin Wansorra*[2,4], *Ralph Steininger*[2], *Benjamin Peerless*[1], *Lothar Weinhardt*[2,3,4], *Clemens Heske*[2,3,4], *Stefanie Dehnen*[1,]*

[1]Institute of Nanotechnology (INT) and Karlsruhe Nano Micro Facility (KNMF), Karlsruhe Institute of Technology (KIT), Kaiserstraße 12, 76131 Karlsruhe, Germany

[2]Institute for Photon Science and Synchrotron Radiation (IPS), Karlsruhe Institute of Technology (KIT), Kaiserstraße 12, 76131 Karlsruhe, Germany

[3]Institute for Chemical Technology and Polymer Chemistry (ITCP), Karlsruhe Institute of Technology (KIT), Kaiserstraße 12, 76131 Karlsruhe, Germany

[4]Department of Chemistry and Biochemistry, University of Nevada Las Vegas (UNLV), Las Vegas, NV 89154-4003, USA

E-mail: stefanie.dehnen@kit.edu

Supporting Information

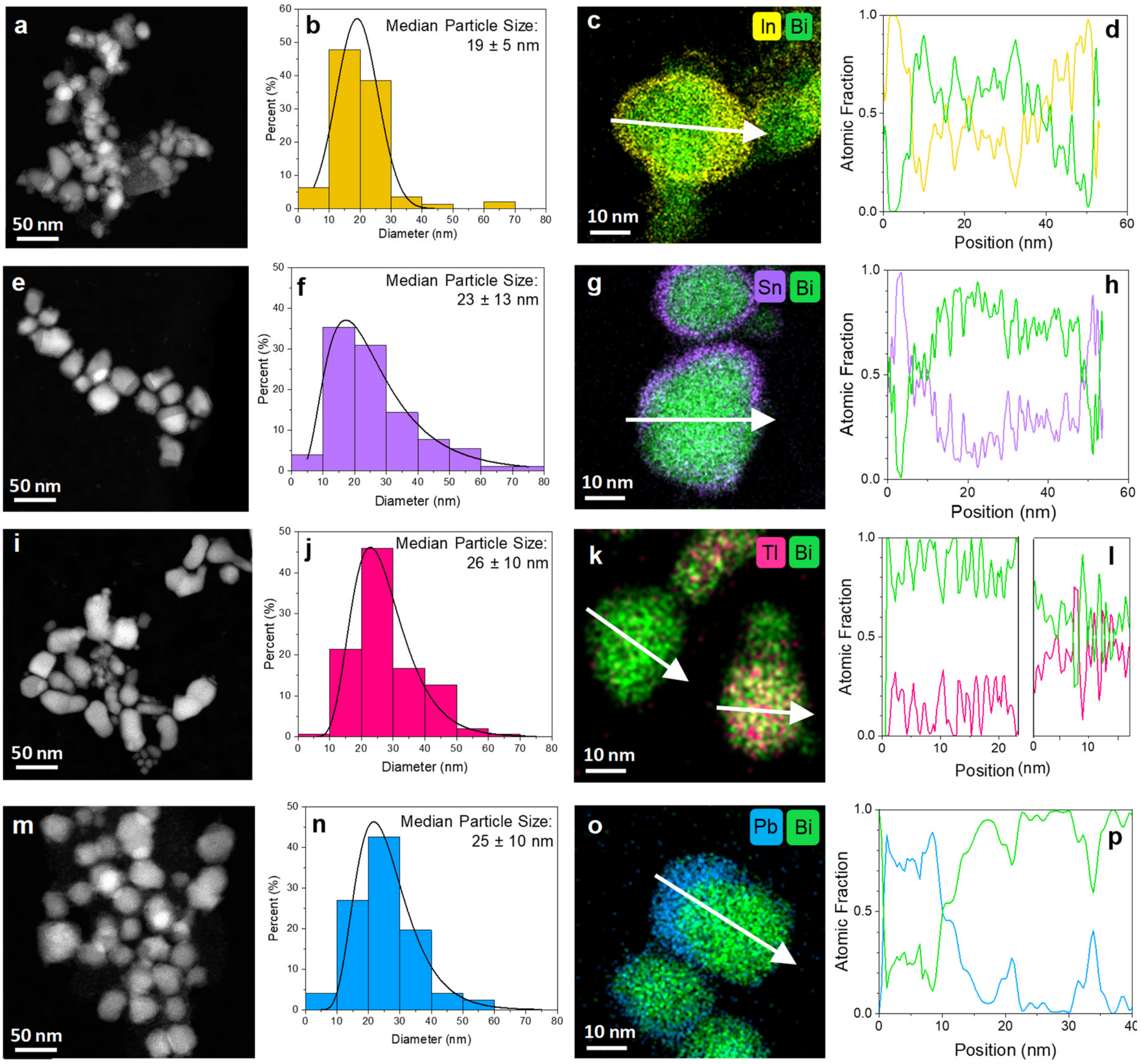

Figure S1. Additional STEM images, particle size distributions, and EDX line scans of the (a-d) In-Bi, (e-h) Sn-Bi, (i-l) Tl-Bi, and (m-p) Pb-Bi nanoparticles. All particle sizes were determined by analysis of TEM images (300 particles counted). Sizes are given as ($\tilde{x} \pm \sigma_{\tilde{x}}$) nm and fitted using a log–normal distribution.

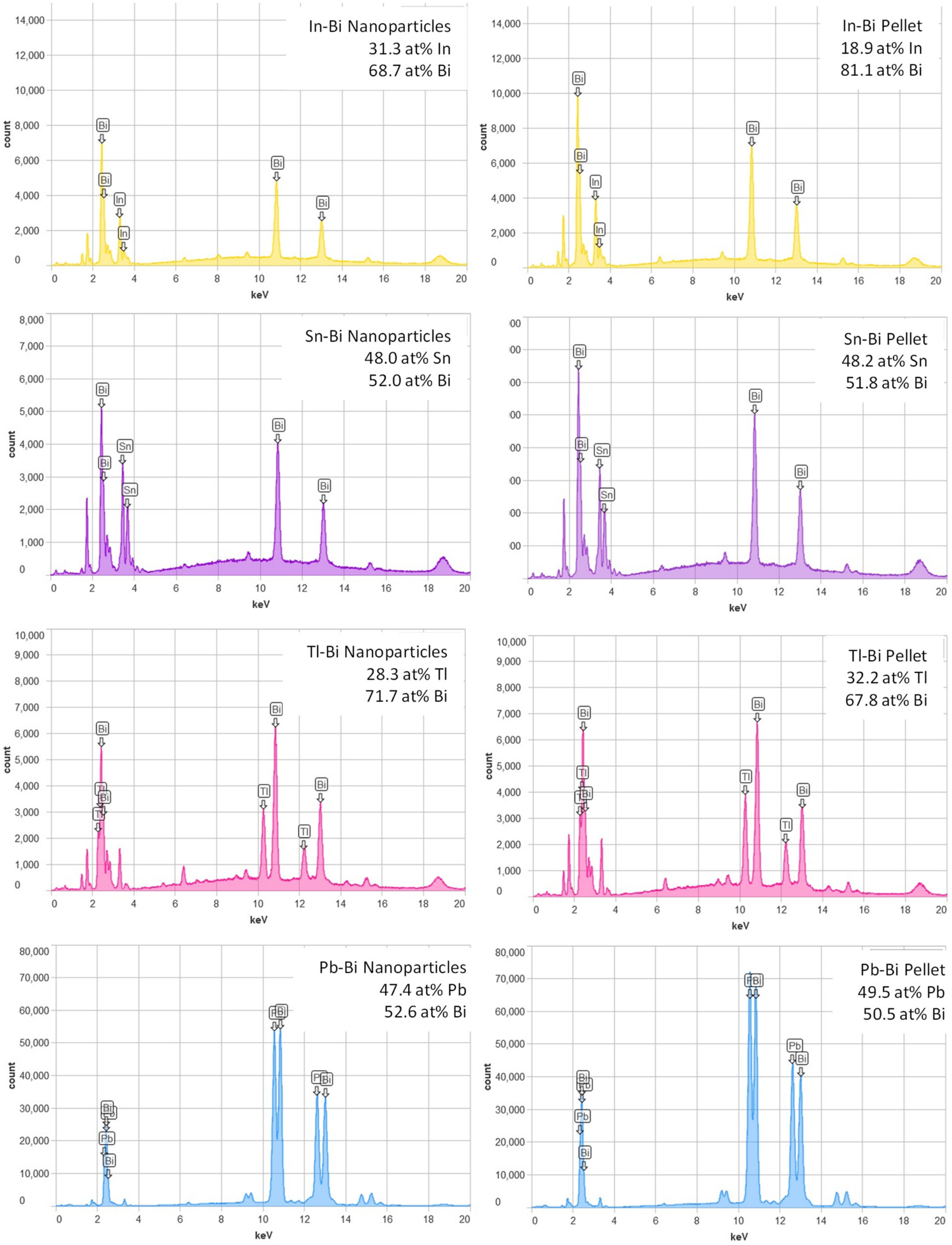

Figure S2. μ-XFS spectra for dried nanoparticle samples and corresponding metal precipitate powders pelleted during the washing process. Insets display calculated M : Bi (M = In, Sn, Tl, or Pb) atomic ratios (in %) for comparison with precursor stoichiometries.

$$D = \frac{K\lambda}{\beta cos\theta}$$

Equation S1. Scherrer Equation for calculating coherent domain lengths (D), where the shape constant K is 1 for spherical nanoparticles, λ is 1.54056 Å for Cu Kα1 radiation, θ is the Bragg diffraction angle, and β is the the full width at half maximum of the peak, in radians.

Table S1. Coherent domain lengths determined by PXRD patterns, calculated using Equation S1 and averaged over the three most intense peaks (for the Bi and Pb_7Bi_3 phases) or the two most intense peaks (for the $TlBi_2$ phase).

Sample	Phase	Reflection	Θ (°)	cosθ	β (radians)	Coherent Domain Length (nm)	Average Coherent Domain Length (nm)
**In-Bi**	**Bi**	(120)	27.185	0.890	0.247	0.702	**0.68**
		(104)	37.989	0.788	0.328	0.596	
		(110)	39.662	0.770	0.265	0.755	
**Sn-Bi**	**Bi**	(120)	27.213	0.889	0.205	0.843	**0.83**
		(104)	37.995	0.788	0.264	0.741	
		(110)	39.739	0.769	0.221	0.908	
**Tl-Bi**	**Bi**	(120)	27.170	0.890	0.091	1.907	**1.95**
		(104)	37.966	0.788	0.114	1.707	
		(110)	39.627	0.770	0.090	2.227	
	**$TlBi_2$**	(110)	31.442	0.853	0.123	1.468	**1.43**
		(160)	32.125	0.847	0.130	1.399	
**Pb-Bi**	**Bi**	(120)	27.177	0.890	0.111	1.557	**1.60**
		(104)	37.959	0.788	0.145	1.348	
		(110)	39.656	0.770	0.105	1.901	
	**Pb_7Bi_3**	(110)	29.407	0.871	0.090	1.969	**1.93**
		(002)	30.829	0.859	0.091	1.981	
		(101)	33.298	0.836	0.101	1.826	

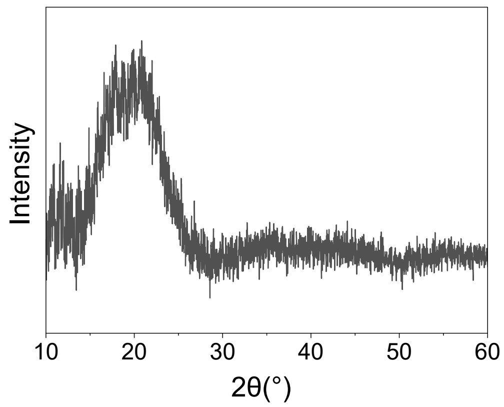

Figure S3. PXRD pattern for PVP, for comparison of nanoparticle samples.

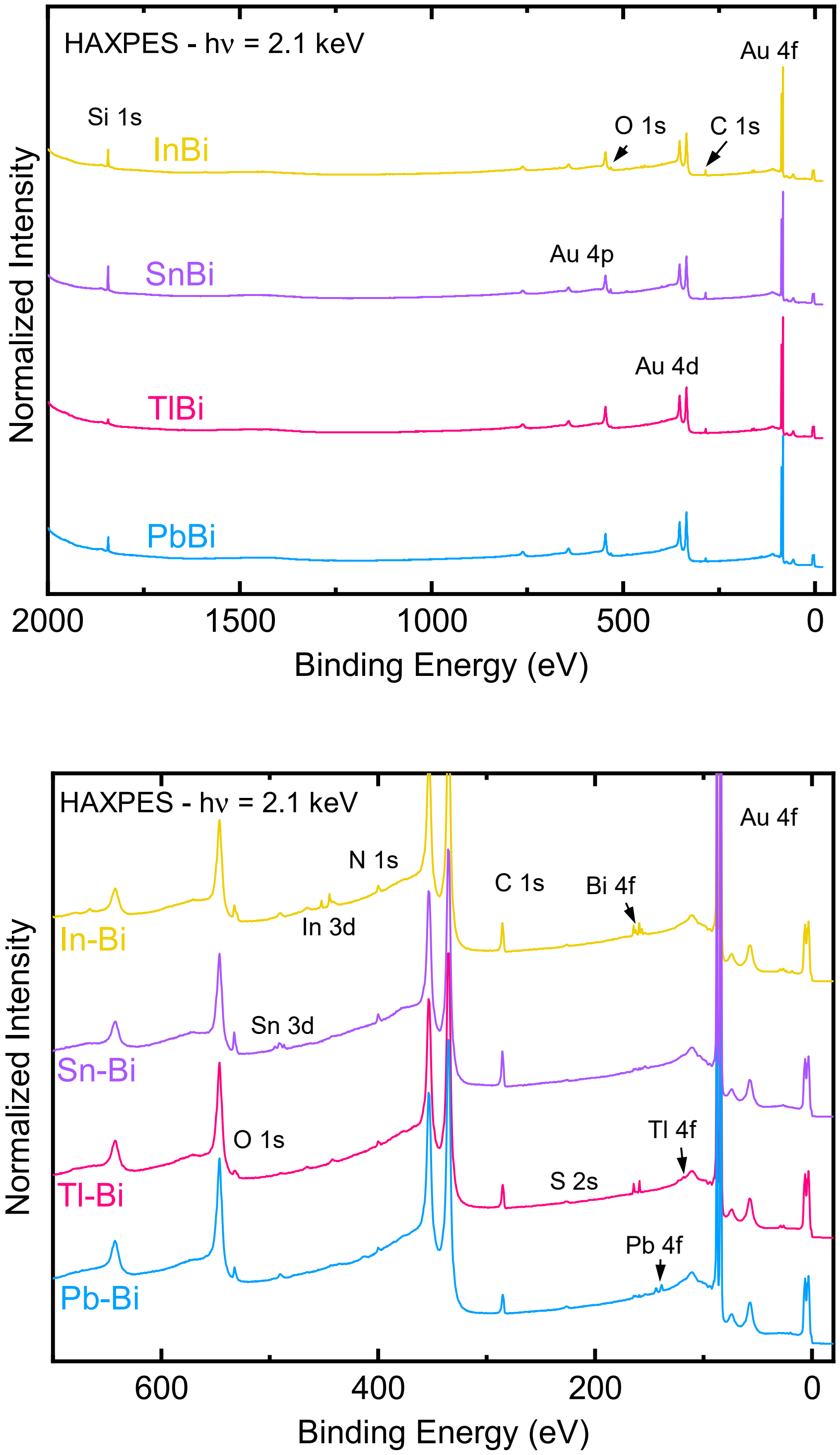

Figure S4. HAXPES spectra (hν = 2.1 keV): survey scan (top) and low-binding-energy region (bottom) for the four nanoparticles samples (top to bottom): In-Bi nanoparticles (yellow), Sn-Bi nanoparticles (purple), Tl-Bi nanoparticles (magenta), and Pb-Bi nanoparticles (blue). Prominent spectral lines are labelled.

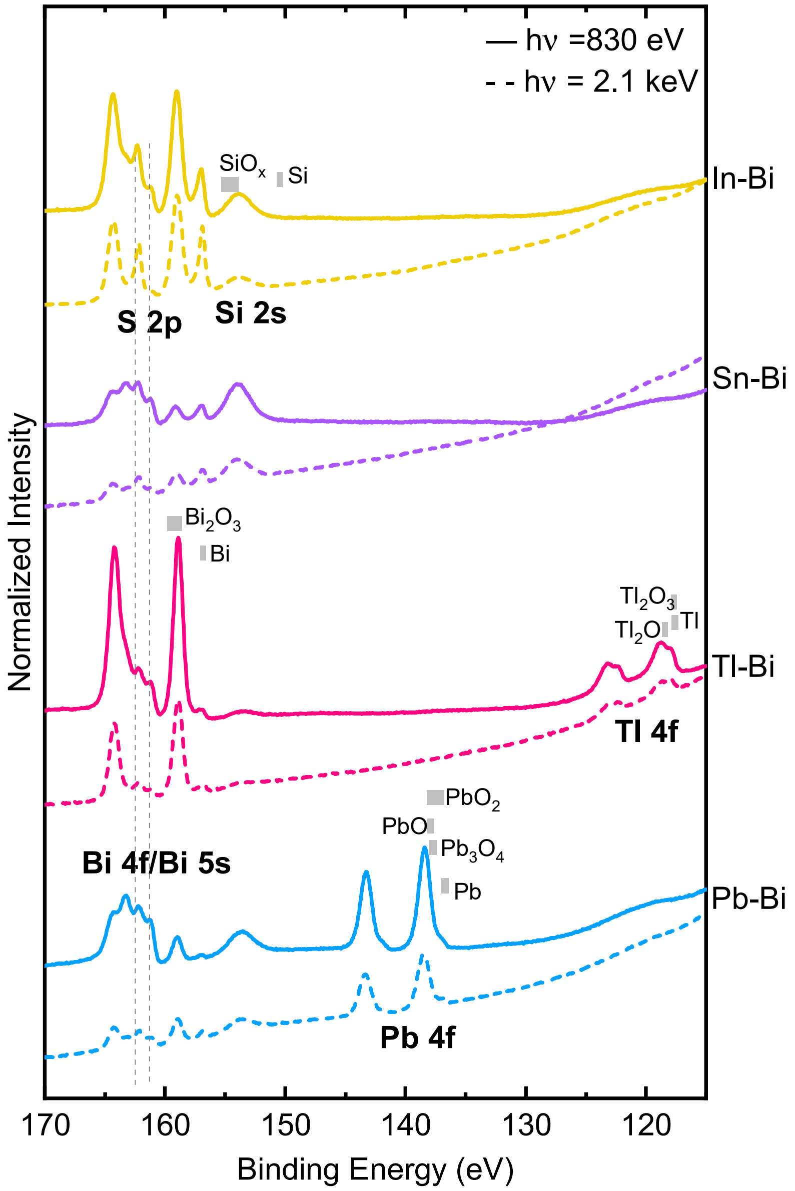

Figure S5. PES (hν = 830 eV, solid line) and HAXPES (hν = 2.1 keV, dashed line) spectra of the Bi 4f, Bi 5s, Pb 4f, Tl 4f, Si 2s, and S 2p region (top to bottom): In-Bi nanoparticles (yellow), Sn-Bi nanoparticles (purple), Tl-Bi nanoparticles (magenta), and Pb-Bi nanoparticles (blue). Literature binding-energy ranges for selected bulk compounds are shown as gray bars above the spectra.

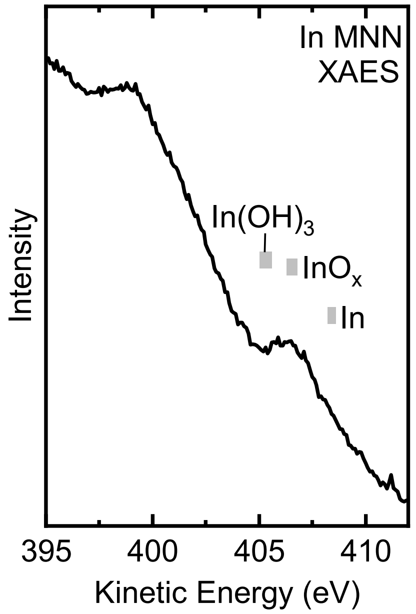

Figure S6. X-ray-excited Auger electron spectroscopy (XAES) spectra of the In MNN region with selected literature binding energies of relevant bulk compounds plotted as gray bars above the spectra.[25]

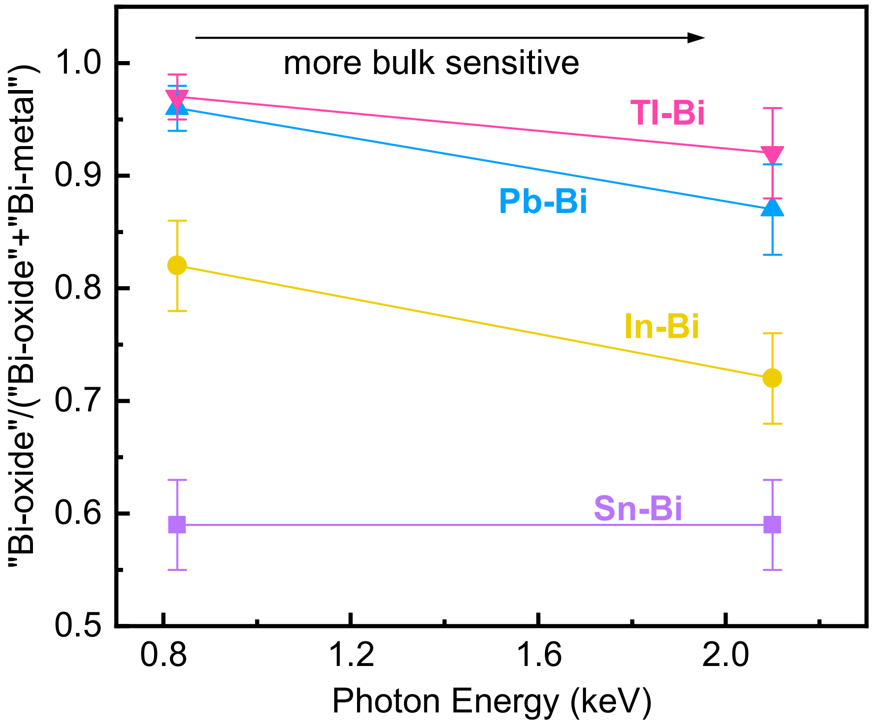

Figure S7. Peak-area ratios of "Bi-oxide"/("Bi-oxide" + "Bi-metal") for PES (hν = 830 eV) and HAXPES (hν = 2.1 keV). Peak areas were determined using the Bi $4f_{7/2}$ core-level peaks.

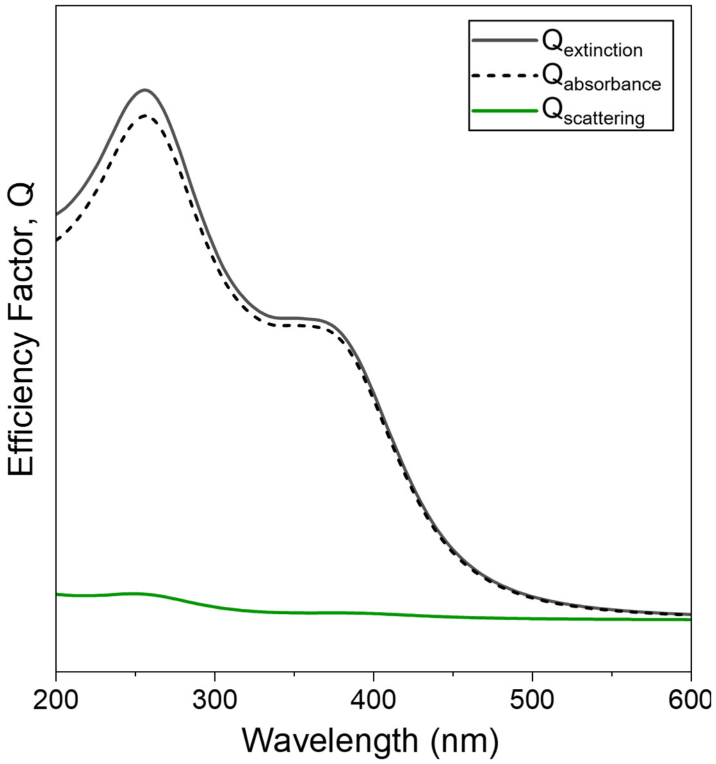

Figure S8. Simulated efficiency factors $Q_{extinction}$, $Q_{absorbance}$, and $Q_{scattering}$ for 20 nm spherical Bi nanoparticles in isopropanol, calculated by Mie theory.[49,50]
